# Educational attainment and anxiety in middle-aged and older Europeans

**DOI:** 10.1038/s41598-023-40196-4

**Published:** 2023-08-16

**Authors:** Adam Chlapecka, Katrin Wolfová, Barbora Fryčová, Pavla Cermakova

**Affiliations:** 1 Third Faculty of Medicine, Charles University Prague, 100 00 Prague, Czech Republic; 2 Department of Neurology and Centre of Clinical Neuroscience, General University Hospital and First Faculty of Medicine, Charles University in Prague, 128 21 Prague, Czech Republic; 3 National Institute of Mental Health, 250 67 Klecany, Czech Republic; 4 Second Faculty of Medicine, Charles University Prague, 150 06 Prague, Czech Republic

**Keywords:** Epidemiology, Population screening, Anxiety

## Abstract

We examined the relationship between educational attainment (EA) and anxiety symptoms in a sample of 77,792 individuals (median age = 64 years, 55% female) from the Survey of Health, Ageing and Retirement in Europe. Using logistic regression, we estimated odds ratio (OR) with 95% confidence interval (CI) for the association between EA (7 educational levels based on International Standard Classification of Education) and anxiety symptoms (12 or more points from the shortened 5-item version of the Beck Anxiety Inventory), adjusting for sociodemographic and health-related factors. We further explored whether the relationship varied by region, sex and age group. Independent of sociodemographic and health-related factors, higher levels of EA were associated with lower odds of anxiety symptoms. The magnitude of this association plateaued at first stage of tertiary education (OR 0.40; 95% CI 0.35–0.47, *p* < 0.001). The association was stronger in females, middle-aged individuals and in Central and Eastern Europe while not apparent in Northern Europe. Our findings suggest that individuals with higher education might be protected against anxiety throughout life. The protective effect of education against anxiety symptoms is more pronounced in less egalitarian regions and in females.

## Introduction

The demographic ageing of the population is bringing substantial changes to European societies, as health care systems must adapt to the specific needs of older adults. Their mental health is becoming an essential concern in medicine^1^. Anxiety disorders constitute the largest group of mental disorders in most Western societies and represent a principal cause of disability^2,3^. Anxiety can generally be described as an unpleasant state of inner turmoil and includes subjectively unpleasant feelings of dread over anticipated events^4^. Most anxiety disorders occur in childhood, adolescence, or early adulthood^5^ but are also common in late adulthood^6^. Approximately 25% of the population is expected to have or have previously had an anxiety disorder^7^. In addition to other contributing factors, such as loneliness or perceived isolation, stigmatization of affected individuals may play a role in the high prevalence of anxiety^8^. Stigmatization refers to the perception of negative attributes by oneself or others and is strongly associated with anxiety and depression^9^. Stigmatization of individuals with mental illness leads to adverse consequences, including delays or avoidance of seeking help, non-adherence to treatment, strained social interactions, reduced quality of life, and diminished self-esteem^10^. There may be cultural differences in the perception of anxiety^11^, such as that on one hand, some cultures may view anxiety as a kind of weakness or personal failing, causing individuals to suppress their symptoms. On the contrary, other cultures may have obtained more open attitude towards anxiety, accepting it and providing necessary supportive systems^11^. Because anxiety disorders are associated with a significant level of disability, high health care utilization and a substantial economic burden on society^12^, they require attention from epidemiological research and public health resources. In epidemiological studies, Beck Anxiety Inventory scale (BAI) is often used to identify individuals with anxiety symptoms, with the aim of quantifying and comparing their occurrence among different populations^13^. Although occasional manifestation of anxiety symptoms is a common part of stressful situations in life, in cases where these symptoms persist and cause substantial impairment in daily functioning, they become an integral feature of anxiety disorders^14^.

Educational attainment (EA) is a strong social indicator of adult health and longevity in European countries^15^. An increase in EA at the population level over time has been related to lower mortality rates^16^. At an individual level, higher EA has been linked to a lower risk of several somatic diseases including dementia^17^, diabetes, heart disease^18^ or cancer^19^. Low EA level has been found to be related to several mental disorders including anxiety and depression^20^. EA might protect against mental disorders through several mechanisms, including greater access to resources, such as rewarding jobs, economic stability, richer social networks, higher socioeconomic status, healthier lifestyle and access to health care^21,22^. EA could also equip individuals with better coping mechanisms, which are independent of sociodemographic and health-related factors^20^.

The majority of studies investigating the potentially protective effects of EA on mental health have consistently reported minimal limitations to the positive impacts associated with EA, suggesting that the association of EA with mental health may be linear^23^. This could be translated into a dose-dependent pattern of EA protection and would imply that there is no upper threshold of the EA benefits on mental health. However, it has been argued that the effect of EA on mental health could diminish in individuals with the highest EA^23,24^, which would suggest that there is an upper limit, beyond which additional EA no longer provides more protection. One of the possible explanations of this phenomenon is overeducation, which is conventionally perceived as a disparity between job requirements and educational qualifications^25^. Specifically, it indicates a situation where the degree of EA acquired surpasses its degree required to adequately fulfil a job. Highly educated individuals are prone to being overqualified for their jobs, which is related to the occurrence of mental health problems^26^.

The protective effect of EA might vary across sociodemographic groups. There is robust evidence on sex differences in the prevalence of anxiety in the population^27,28^. For instance, a European population-based epidemiological study conducted by Tetzner et al. has shown that females tend to report higher levels of anxiety compared to males^29^. McLean et al. in their observational study on the U.S population demonstrated that probability to develop anxiety disorders is almost two times higher for females in comparison to males^27^. Females had historically fewer opportunities for schooling and attained lower educational levels than males^30^, and given the differences in the association of EA with mental health outcomes based on sex ^31^, it is plausible that sex may moderate the relationship between EA and anxiety. Although previous research demonstrated that age is strongly associated with the prevalence of anxiety in the population^28^, there are inconsistent results regarding how the protective effect of EA on mental health is influenced by age. According to Bjelland, the advantages of EA increase over the course of the lifetime^20^. On the contrary, our prior work indicates that EA provides greater mental health protection to individuals under the age of 65^24^.

Furthermore, several studies indicate a differential effect of EA on health outcomes across regions. For example, when compulsory schooling was introduced in the United States, it reduced mortality^32^, but this effect was not observed in France^33^ or England^34^. One potential explanation for this phenomenon could be attributed to the presence of more egalitarian societies, stronger social security systems, and the implementation of preventive measures for mental health across multiple sectors in European countries. Consequently, the impact of EA alone in mitigating mortality rates might be less pronounced in comparison to the United States. Another European study by Gathmann et al. supports this variability and finds large benefits of compulsory education for instance in Belgium, but not in Spain^35^. Even though some explanations for the differences between countries exist, the mechanisms, which underlie them, are still largely unknown.

The goals of our study are to (1) investigate the relationship between EA and anxiety symptoms while taking into account various sociodemographic and health-related factors; (2) identify a potential dose-dependence of this association, or on the contrary, an upper limit, above which no further profit of EA can be detected, (3) examine regional and demographic discrepancies in the association between EA and symptoms of anxiety.

## Materials and methods

### Source of data

The current study used data from Survey on Health, Ageing and Retirement in Europe (SHARE), as described in another publication in detail^36^. SHARE is a large multidisciplinary, longitudinal study of the European population. Individuals who are at least 50 years of age and their partners, irrespective of age, answer survey questions by a computer-assisted personal interview (CAPI). Data are collected biennially. The first wave of SHARE took place in 2004 with 30,000 respondents in 11 European countries. Additional countries participated in subsequent seven waves that were conducted in approximately 2-years intervals. SHARE has been repeatedly reviewed and approved by the University of Mannheim Ethics Committee (waves 1–4) and the Ethics Council of the Max Plank Society (waves 4–7), Ethical Approval Reference Number 2021_24. All methods were performed in accordance with the relevant guidelines and regulations. An informed consent was signed by every participant.

### Educational attainment

We categorized EA into seven levels according to International Standard Classification of Education 1997 (ISCED-97)^37^: no or pre-primary level of education (level 0), primary level of education (level 1), lower secondary level (level 2), upper secondary level (level 3), post-secondary non-tertiary education (level 4), first stage of tertiary education (level 5) and second stage of tertiary education (level 6). Distribution of sex and age across different EA levels showed highest proportion of females in EA level 1 (61%) and highest median age in EA level 1 (73 years; not presented in tables.)

### Anxiety symptoms

Anxiety symptoms were measured by five items, which are part of the Beck Anxiety Inventory (BAI)^13^, including one item related to psychological symptoms (“*I had fear of worst happening”),* two items related to physiological symptoms (“*I felt my hands trembling”* and* “I felt faint”),* and two items related to cognitive symptoms (“*I was nervous*” and “*I had a fear of dying*”). The scale reached an acceptable level of internal consistency (Cronbach alfa coefficient 0.71). Each item was scored on a four-point Likert scale (1 = “never”, 2 = “hardly ever”, 3 = “some of the time”, 4 = “most of the time”) and the total score was the sum of the individual scores. As a clinical screening tool, the BAI provides cut-off scores estimating the severity of anxiety symptoms. Based on the distribution of anxiety in the previous work using SHARE data^29^, we used the 90th percentile of the score (cut- off 12 or more points) to indicate severe anxiety symptoms, referred to hereafter as “anxiety symptoms”. To test the robustness of this approach, we have conducted sensitivity analyses with the 95th percentile (cut- off 14 or more points).

### Covariates

Based on previous literature^38–40^, we identified several socioeconomic and health-related characteristics associated with EA and anxiety symptoms, which serve as confounders in our analysis. All the covariates were recorded in the wave when anxiety symptoms were assessed or in the closest wave. If data were missing for a certain characteristic in the particular wave, the value from the closest wave was used instead. There was less than 5% of missing data in the final sample. Descriptive analysis also includes individuals with missing data, but multivariable analysis was performed only on complete cases. Sociodemographic characteristics were age (in years), sex (female vs. male), household net worth (standardized difference between household gross financial assets and financial liabilities), number of children and grandchildren, family status (living with a partner vs. alone), employment status (working vs. not working), household size (number of household members) and neighbourhood (rural vs. non-rural area).

Health-related characteristics were number of limitations in instrumental activities of daily living (IADL), number of chronic diseases (measured by self-reported physicians ‘ diagnosis; including heart disease, stroke, hypertension, diabetes or high blood sugar, cancer, lung disease, and general disability), body mass index (BMI), physical limitation measured by mobility limitation index, cognitive status based on 10 words delayed recall test, depressive symptoms measured by EURO-D scale, maximal grip strength (an indicator of overall physical fitness and capacity, used in research and clinical setting^41^, associated with deterioration of mental health, including vulnerability to depression, anxiety or cognitive decline^42,43^), physical inactivity (never vigorous nor moderate physical activity vs. physical activity), smoking (ever smoked daily vs. never smoked daily), alcohol use (drinking more than 2 glasses of alcohol per day vs. drinking less), frequency of eating fruits or vegetables per day, drugs against depression or anxiety and drugs against sleep problems.

### Study sample

**Figure 1:**
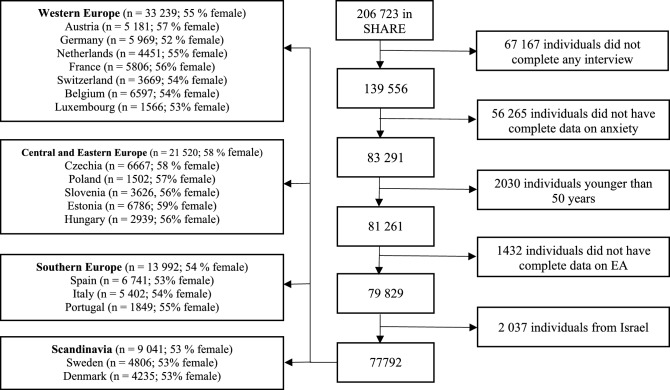
Selection of the study sample.

As anxiety was measured in the fourth and fifth wave of SHARE, we used these for the analyses in the present study. We used solely the observations from the wave, in which the participants had complete data on anxiety symptoms for the first time. Thus, when individuals had complete data in both waves four and five, we only considered the data from wave four for the analysis. Data on covariates were from the same wave. If information on covariates was not available from the same wave as anxiety symptoms, data from the closest wave were used. From the 139,556 participants who completed at least one interview in SHARE, we excluded those who did not have complete data on anxiety symptoms (n = 56,265), were younger than 50 years of age (n = 2030) and had missing data on EA (n = 1432). Participants from Israel were also excluded as this analysis focused on the European population (n = 2037). The final analytical sample consists of 77,792 participants from 4 European regions: Western Europe (n = 33,239 including Austria, Germany, Netherlands, France, Switzerland, Belgium, Ireland and Luxembourg), Central and Eastern Europe (CEE, n = 21,520 including Czech Republic, Poland, Hungary, Slovenia, Estonia and Croatia), Southern Europe (n = 13,992 including Spain, Italy, Greece, Portugal) and Northern Europe (n = 9041 including Sweden and Denmark). Flowchart is presented on Fig. 1.

### Statistical analysis

Descriptive characteristics of the sample are presented as frequency (n, %), mean ± standard deviation (SD), or median and interquartile range (IQR), where appropriate. Differences in all measurements between individuals with and without anxiety symptoms were compared using independent samples t-test, Mann–Whitney test and Chi-square test, where appropriate. Subsequently, logistic regression was conducted to estimate odds ratio (OR) with 95% confidence interval (CI) for the association of EA (level 0 as reference) with anxiety symptoms, step-wise adjusting for sociodemographic and health-related characteristics. Model 1 was adjusted for sex and age, Model 2 also for the remaining sociodemographic characteristics and Model 3 also for health-related characteristics. The model fit was assessed with Akaike Information Criterion. In order to evaluate potential multicollinearity between covariates, we used variance inflation factor (VIF). All covariates had VIF < 3 and were thus kept in the analysis.

Due to considerable differences in both EA^44^ and the burden of anxiety disorders across European regions^2^, we investigated whether there were regional differences in the association of EA with anxiety symptoms. Thus, a two-way interaction term between EA and region was added into Model 1 and the interaction effect was evaluated with a likelihood ratio (LR) test. Due to the insufficient number of participants for each EA level in each region, the original seven levels of EA were joint into three groups: low education (levels 0 and 1), middle education (levels 2–4), and high education (levels 5 and 6). All three models were stratified by region. We tested age and sex as potential moderators because these factors have been frequently reported to moderate the association of EA with mental health^20^. To assess the moderating effect of sex and age group (younger than 65 years vs. older than 65 years) on the association of EA with anxiety symptoms, we included an interaction term between sex/age group and EA in Model 1. We used likelihood ratio (LR) test to measure the effect of interaction and performed stratified analyses, where appropriate.

In addition, as socioeconomic position of females differs considerably across European regions^45^ and as it was suggested that females may gain a larger benefit from education than males in mitigating health-related risks^46^, we performed a sensitivity analysis, in which we specified multilevel logistic regression models with random intercept set at the country level and an interaction term between sex and EA. We used LR test to assess the effect of interaction and intraclass correlation coefficient (ICC) to evaluate the variance in the outcome variable that is explained by the grouping structure. We performed all statistical analyses in R software (RStudio Version 1.4.1717).

## Results

**Figure 2:**
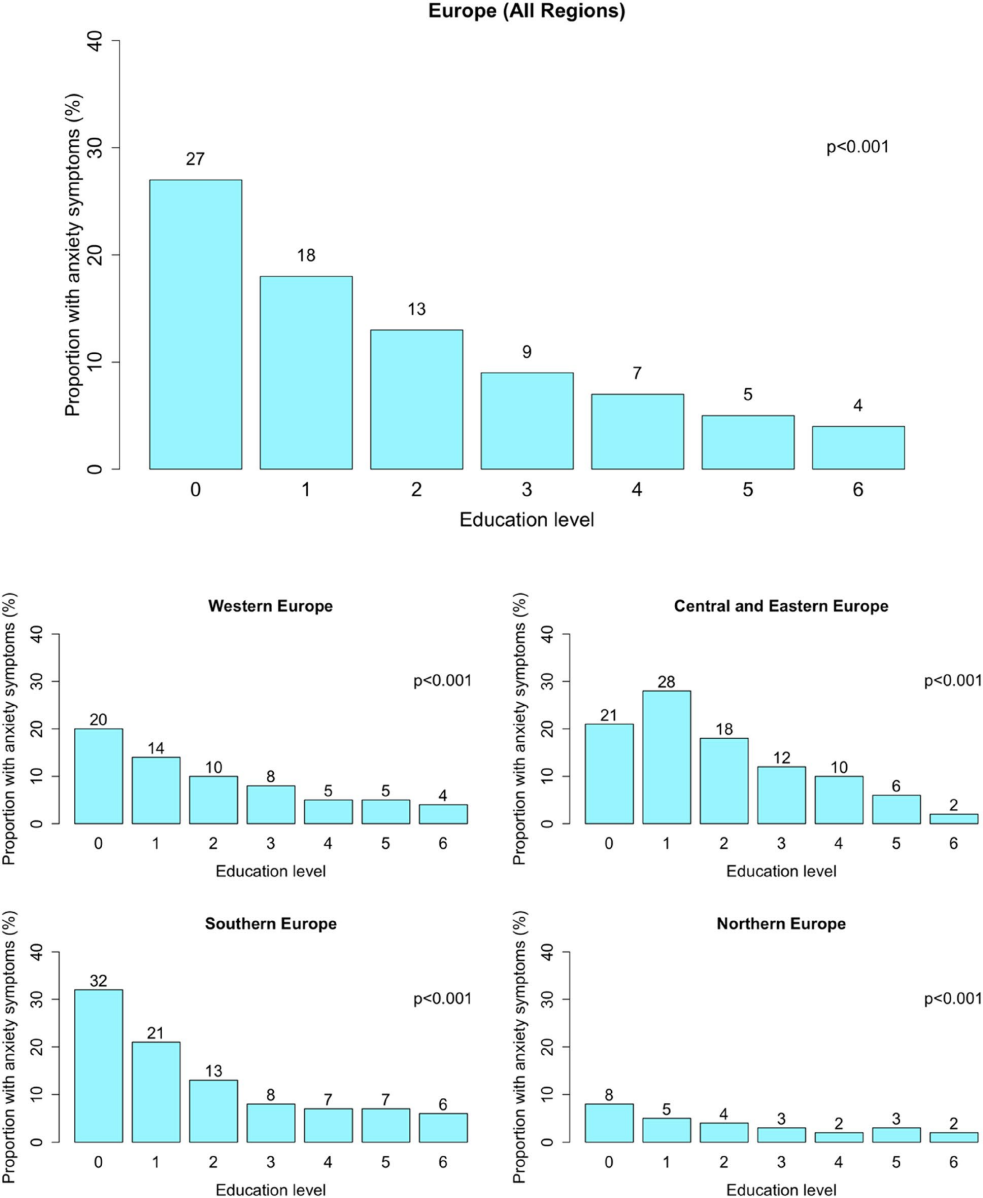
Occurrence of anxiety symptoms in different educational attainment levels.

Among 77,792 participants (median age = 64 years, 55% female), a total of 8638 (11%) presented with anxiety symptoms (cut-off 12 or more points on BAI scale). In general, those with anxiety symptoms were older, more often females, had lower socioeconomic status and a worse health profile (Table 1). Spearman’s correlation between anxiety symptoms and depressive symptoms was 0.50. Correlation matrix between selected health-related characteristics is included in the Supplement (Supplementary Table S.1). The prevalence of anxiety symptoms decreased with increasing EA from 27% in the EA level 0 to 4% in the EA level 6. Region stratification (Fig. 2) showed that anxiety symptoms were most frequent among participants with EA level 0 (highest in Southern Europe: 32%; lowest in Northern Europe: 8%), except for CEE, where the highest prevalence was among participants with EA level 1 (28%). The lowest prevalence of anxiety symptoms was observed in EA level 6 in all regions, although in Northern Europe, it did not considerably differ between EA levels 3–6. Proportion of participants with no or pre-primary level of education, stratified by sex, age and region is included in the Supplement (Supplementary Table S.2).

**Table 1:** Characteristics of participants.

Total number of participants n = 77 792	Anxiety symptoms	
	Yes 8638 (11%)	No 69,154 (89%)
Sociodemographic factors		
Age median (IQR)**	68.00 (19)	64.00 (15)
Female, n (%)**	5823 (67%)	37,135 (54%)
Highest decile of household net worth, n (%)**	359 (4%)	7421 (11%)
Children, one and more, n (%)	7763 (90%)	62,589 (91%)
Grandchildren, one and more, n (%)**	6374 (74%)	45,802 (66%)
Living with a partner, n (%)**	5549 (64%)	51,690 (75%)
Currently working, n (%)**	1020 (12%)	20,871 (30%)
Household size median (IQR)**	2.00 (0.00)	2.00 (1.00)
Living in a rural area, n (%)*	2654 (32%)	22,209 (33%)
Health-related factors		
At least 1 limitation in IADL, n (%) **	3825 (44%)	9239 (13%)
Number of chronic diseases, median (IQR) **	2.00 (3.00)	1.00 (1.00)
BMI, mean ± SD **	27.41 (5.36)	26.76 (4.58)
Mobility limitations index, median (IQR) **	3.00 (5.00)	0.00 (2.00)
Delayed recall, median (IQR)**	3.00 (3.00)	4.00 (2.00)
Number of depressive symptoms, median (IQR)**	5.00 (4.00)	2.00 (2.00)
Maximal grip strength, mean ± SD**	27.59 (11.33)	34.68 (11.93)
Physical activity, n (%)**	5847 (68%)	62,877 (91%)
Never smoked daily, n (%)**	4021 (47%)	27,645 (40%)
No excessive drinking, n (%)**	7535 (88%)	56,753 (82%)
Frequency of eating fruits and vegetables per day, median (IQR)**	1.00 (1.00)	1.00 (0.00)
Drugs for anxiety/depression, n (%)**	1964 (23%)	4241 (6%)
Drugs for sleep problems, n (%)**	1944 (23%)	4686 (7%)

**p < 0.001; *p < 0.05; BMI, body mass index; IADL, instrumental activities of daily living; SD, standard deviation; IQR, interquartile range; *p*-values were derived from independent samples t-tests, Mann–Whitney U tests and Chi-square tests.

Higher levels of EA were associated with lower odds of anxiety symptoms (Table 2, Model 1). Adjustment for sociodemographic and health-related covariates decreased the strength of the association between EA and anxiety symptoms, however, it remained statistically significant in all models (Table 2, Model 3). When adjusted for all covariates, all higher levels of EA were associated with lower odds of anxiety symptoms in comparison to EA level 0 (Table 2, Model 3). The magnitude of this association had a dose–response pattern from EA level 1 (OR 0.68; 95% CI 0.60–0.78), through level 5 (OR 0.40; 95% CI 0.35–0.47), however, it did not further increase in the EA level 6 (OR 0.44; 95% CI 0.27–0.67).

**Table 2:** Association of educational attainment with anxiety symptoms.

EA	Model 1	Model 2	Model 3
Level 0	*Reference*	*Reference*	*Reference*
Level 1	0.58 (0.53; 0.63)**	0.57 (0.52; 0.63)**	0.68 (0.60; 0.78)**
Level 2	0.44 (0.40; 0.48)**	0.43 (0.39; 0.48)**	0.59 (0.52; 0.67)**
Level 3	0.31 (0.28; 0.34)**	0.33 (0.30; 0.36)**	0.54 (0.48; 0.62)**
Level 4	0.24 (0.21; 0.28)**	0.27 (0.23; 0.32)**	0.44 (0.36; 0.54)**
Level 5	0.16 (0.15; 0.18)**	0.21 (0.19; 0.23)**	0.40 (0.35; 0.47)**
Level 6	0.14 (0.10; 0.21)**	0.22 (0.14; 0.32)**	0.44 (0.27; 0.67)**
*AIC*	*51,275*	*47,967*	*32,440*

Results are odds ratio with 95% confidence intervals derived from logistic regression for the association of educational attainment with anxiety symptoms.

AIC, Akaike information criterion.

Model 1: age, sex.

Model 2: age, sex, household net worth, employment status, family status, number of children, number of grandchildren, household size, area of living.

Model 3: age, sex, household net worth, employment status, family status, number of children, number of grandchildren, household size, area of living, limitations in instrumental activities of daily living, 10 words delayed recall test, number of chronic diseases, , body mass index, mobility limitation index, number of depressive symptoms, maximal grip strength, physical activity, smoking, excessive alcohol intake, frequency of eating fruits and vegetables per day, drugs for anxiety/depression, drugs for sleep problems.

**p < 0.001; *p < 0.05.

We observed a significant interaction (*p* value from LR test < 0.001 in Model 1) between EA and region. In all regions, both middle and high education were associated with lower odds of anxiety symptoms when compared to low education in age–sex adjusted models (Table 3, Model 1). In the fully adjusted model, the greatest association was present in CEE (OR for high vs. low education 0.49; 95% CI 0.40–0.61). On the contrary, the association in Northern Europe disappeared in the fully adjusted model (OR for high vs. low education 1.04; 95% CI 0.67–1.63). Both sex (*p* from LR test 0.02 in Model 1) and age group (*p* from LR test 0.01 in Model 1) were found to be effect modifiers in the association between EA and anxiety symptoms. A stronger association between anxiety symptoms and EA could be observed in females, when compared to males, and in younger, relative to older individuals (Table 4).

**Table 3:** Association of educational attainment with anxiety symptoms stratified by European region.

	Model 1	Model 2	Model 3
*Low Education*	*Reference*	*Reference*	*Reference*
Western Europe			
Middle education	0.60 (0.54; 0.65)**	0.65 (0.59; 0.72)**	0.97 (0.86; 1.10)
High education	0.34 (0.30; 0.39)**	0.42 (0.37; 0.49)**	0.75 (0.63; 0.88)**
Southern Europe			
Middle education	0.51 (0.46; 0.57)**	0.56 (0.49; 0.62)**	0.68 (0.58; 0.79)**
High education	0.31 (0.24; 0.38)**	0.39 (0.31; 0.50)**	0.51 (0.37; 0.68)**
Central and Eastern Europe			
Middle education	0.53 (0.48; 0.59)**	0.58 (0.52; 0.65)**	0.69 (0.60; 0.80)**
High education	0.22 (0.19; 0.26)**	0.32 (0.26; 0.38)**	0.49 (0.40; 0.61)**
Northern Europe			
Middle education	0.68 (0.50; 0.94)*	0.72 (0.52; 1.00)*	0.95 (0.65; 1.42)
High education	0.50 (0.35; 0.72)**	0.61 (0.42; 0.88)*	1.04 (0.67; 1.63)

Results are odds ratio with 95% confidence intervals derived from logistic regression for the association of educational attainment with anxiety symptoms.

AIC, Akaike information criterion.

Model 1: age, sex.

Model 2: age, sex, household net worth, employment status, family status, number of children, number of grandchildren, household size, area of living.

Model 3: age, sex, household net worth, employment status, family status, number of children, number of grandchildren, household size, area of living, limitations in instrumental activities of daily living, 10 words delayed recall test, number of chronic diseases, , body mass index, mobility limitation index, number of depressive symptoms, maximal grip strength, physical activity, smoking, excessive alcohol intake, frequency of eating fruits and vegetables per day, drugs for anxiety/depression, drugs for sleep problems.

**p < 0.001; *p < 0.05.

**Table 4:** Association of educational attainment with anxiety symptoms in the whole analytical sample stratified by sex and age group.

	Male	Female	Age 50–64 years	Age 65 years or older
Low education	Reference			
Model 1				
Middle education	0.58 (0.53–0.64)**	0.52 (0.49–0.55)**	0.46 (0.43–0.51)**	0.53 (0.50–0.56)**
High education	0.29 (0.26–0.34)**	0.23 (0.21–0.26)**	0.21 (0.18–0.23)**	0.26 (0.23–0.29)**
Model 2				
Middle education	0.62 (0.57–0.68)**	0.55 (0.51–0.58)**	0.55 (0.50–0.60)**	0.56 (0.52–0.60)**
High education	0.39 (0.34–0.45)**	0.30 (0.27–0.33)**	0.32 (0.28–0.37)**	0.32 (0.29–0.36)**
Model 3				
Middle education	0.78 (0.69–0.88)**	0.75 (0.69–0.81)**	0.72 (0.64–0.80)**	0.79 (0.72–0.86)**
High education	0.60 (0.50–0.71)**	0.52 (0.45–0.60)**	0.53 (0.45–0.62)**	0.57 (0.49–0.66)**

Results are odds ratio with 95% confidence intervals derived from logistic regression for the association of educational attainment with anxiety symptoms.

AIC, Akaike information criterion.

Model 1: age, sex.

Model 2: age, sex, household net worth, employment status, family status, number of children, number of grandchildren, household size, area of living.

Model 3: age, sex, household net worth, employment status, family status, number of children, number of grandchildren, household size, area of living, limitations in instrumental activities of daily living, 10 words delayed recall test, number of chronic diseases, , body mass index, mobility limitation index, number of depressive symptoms, maximal grip strength, physical activity, smoking, excessive alcohol intake, frequency of eating fruits and vegetables per day, drugs for anxiety/depression, drugs for sleep problems.

**p < 0.001; *p < 0.05.

### Sensitivity analyses

Variance in anxiety symptoms was not substantially influenced by the grouping structure in multilevel models with country-specific random intercept. In Model 1, only 12% of the variation in anxiety symptoms was due to between-country differences, which decreased to only 10% and 8% in Model 2 and Model 3, respectively (not presented in tables). The interaction between sex and EA was significant (p from LR test 0.01 in Model 1). In stratified Models 1 and 2, EA levels 1–2 were associated with higher odds of anxiety symptoms in females than males, whereas EA levels 3, 4 and 5 were associated with more anxiety symptoms in males (not presented in tables). In the fully adjusted model, female sex was associated with more anxiety symptoms in all EA levels (not presented in tables).

When using a higher cut-off on BAI (95th percentile, cut-off 14 or more points), we observed a smaller magnitude of the association, but similar results. Higher EA was still associated with lower odds of anxiety symptoms, except for EA level 6, where the association lost statistical significance (not presented in tables).

## Discussion

Based on this large population-based cohort study capitalizing on nearly 78,000 middle-aged and older Europeans from 17 countries, we found that higher levels of EA were associated with lower odds of anxiety symptoms. This association was not explained by participants´ sociodemographic and clinical characteristics and was larger in females and younger individuals. The gradient of anxiety symptoms driven by EA was greatest in CEE and weakest in Northern Europe.

Several previous studies suggest a bi-directional association of EA and anxiety^20,26,47–50^. On one hand, education might provide psychosocial resources, such as greater control, cognitive skills and self-efficacy that may lead to higher resilience with regards to strain and facilitate ways how to cope with stressors in order to diminish anxiety^20,47^. Higher EA together with higher resilience could lead to a greater social and economic capital, more fulfilling jobs, and wider occupational options, which protect against the onset and/or exacerbation of worries and fears^5,20^. On the other hand, there is a strong evidence that early-childhood stressful events alter hypothalamic pituitary adrenal axis, thereby increasing children’s susceptibility to anxiety ^51,52^. This may further impose a barrier to completing education, at least partly mediated by high levels of nervousness during exams and in-class presentations^50^. Evidence shows that individuals with anxiety disorders have substantially impaired academic performance throughout the formative years and are at risk of school dropout^48,50^.

Tambs et al. suggest that the relationship might be also determined by shared genetic factors that lead to both lower educational level and anxiety^49^. However, there are other studies, which did not detect any association between EA and anxiety disorders^20,22^. Despite the fact that cross-sectional design of our study is not able to resolve the issue of directionality, it can give an insight into the question whether the association between EA and anxiety symptoms is direct or mediated by other factors. Although the association attenuated when we adjusted for a number of participants´ characteristics, it remained significant even in the fully adjusted models. Hence, we suggest there exists a direct relationship between EA and anxiety symptoms.

Prior research documented a threshold for the level of EA, above which further years of education are no longer protective for mental health^24^. Additional years of education were found to not provide further protection against depressive symptoms after the first stage of tertiary education in one study, and against affective disorders after secondary education with high school graduation in another study^22,24^. It is less clear, whether such threshold exists in the context of anxiety disorders. Our results are in line with these studies, suggesting that the relationship between anxiety symptoms and EA follows a dose response pattern with a threshold at the level of the first stage of tertiary education. This finding may be explained by previously proposed theory, that additional EA above certain threshold may not offer any extra protection for mental health when people with the highest education are not able to find a suitable occupation to match their expertise^26^.

We found a similar pattern in males and females as well as in younger and older individuals, however, there were differences in the strength of the association. In line with the view that the benefits of EA on mental health are greater for females^31^, our results suggest that higher EA might provide greater protection against anxiety symptoms in females than males. Further sensitivity check showed better protection in females after completion of upper secondary education or higher degrees. These results are supported by similar findings from studies on the relationship between education and depression^31^. We interpret these findings as support for the theory of resource substitution, which claims that since females have fewer social and economic resources at their disposal, education can fill in these gaps, making the lack of other resources less harmful, which potentiates the benefit of EA on mental health^31^. We also found that the association between EA and anxiety symptoms is stronger in individuals younger than 65 years relative to those aged 65 and older. We argue that mental health of older adults may be less influenced by the benefits of education, such as control, cognitive skills or self-efficacy, because these skills may lose their value in old age. Alternatively, the quality of education may have increased over time, so more recent cohorts could benefit from health promoting effects of EA to a larger extent than older cohorts.

Similarly to previous research on EA in relation to depressive symptoms^24^, we found the strongest association between EA and anxiety symptoms in CEE, while the relationship was weakest in Norther Europe. The impact of EA on anxiety symptoms or, the other way around, the impact of anxiety symptoms on EA may be greater in CEE because countries in these regions do not have strong preventive policies that could mitigate risks for mental disorders, making education an even more powerful determinant of anxiety symptoms. This stands in contrast with countries situated in Northern Europe, where high standards of living conditions and welfare state are in place, which may be the reason why the gradient in anxiety symptoms driven by inequalities in EA is weak.

Several limitations need to be mentioned. This cross-sectional study does not allow us to determine temporality in the association between EA and anxiety symptoms. Residual confounding, evaluating for instance adverse childhood experiences^53^, access to healthcare^54^ or socioeconomic status of the family, in which an individual was raised^55^ is present, which precludes us from establishing causality. Another potential bias arises from cross-sectional estimates based on longitudinal studies (e.g. SHARE) for the inability to account for temporal changes and the dynamic nature of the variables^56^, particularly considering regional changes, as the number of waves and refreshment samples differs across the countries in SHARE. Additionally, we tested only age and sex as potential moderators. However, we acknowledge that the association of EA with anxiety symptoms might vary by other factors, such as income or health status. We believe there is a limitation concerning marital status that we should address. Living single for the whole life might have a different effect on developing anxiety than experiencing widowhood. However, both of these situations can involve a significant change in social support. Living single throughout life may result in the lack of consistent emotional support, which could lead to the higher vulnerability to anxiety. Nevertheless, similar effect could be observed in the situation of losing a partner who may have served as primary source of emotional support and stability.

Furthermore, we acknowledge that the sample is not fully representative to the general population as people with higher education and better health tend to participate in surveys such as SHARE. There are 25% of males and 21% of females 55–74 years old with tertiary education in SHARE compared to 20% of males and 15% of females 55–74 years old with tertiary education in general population based on Eurostat data (collected in the same year as wave 4 of SHARE). This discrepancy may lead to underestimation of the associations we found. Moreover, it has been observed that older respondents with worse health-status are more likely to drop out from the longitudinal study^56^. Another potential bias, which has already been observed in SHARE data, is country-specific discrepancy in reporting of health by education^57^. Generally, older Europeans with higher levels of education tend to evaluate a given health state more negatively and adjusting for these disparities typically leads to increased health inequalities^57^. Notably respondents from Sweden and Denmark demonstrate higher tendency to significantly overestimate their health status^58^. Since we utilized data from either wave four or wave five (when data on anxiety symptoms in wave four was incomplete) of SHARE, the choice of a specific wave could potentially introduce bias in the results. In addition, there is a limitation associated with the measure of anxiety symptoms, which we used as we chose the cut-off arbitrarily. A score of 26–63 points on the original 21-item scale indicates severe (clinically relevant) anxiety symptoms. However, as discussed previously^29^, there is no valid cut-off scores for the shortened 5-item version of BAI. Nevertheless, we performed a sensitivity analysis using a higher cut-off and found similar results.

Despite these limitations, our findings support the notion that targeting preventative strategies to people with lower EA, especially females, has the highest potential to improve mental health of the population in later life. Our results also suggest that the mechanisms of the protective effect of education might be direct, thus improving educational outcomes could also alleviate the burden of mental disorders in Europe.

### Supplementary Information

Supplementary Tables.

## Data Availability

Access to the SHARE data is provided free of charge for scientific use globally upon registration via the SHARE Research Data Center. Further information can be found on the website www.share-eric.eu. The corresponding author of this study will readily share the study protocol and statistical analysis syntax upon request.
