# Supplementary material for: Educational attainment and anxiety in middle-aged and older Europeans
Source: Sci Rep. 2023 Aug 16;13:13314. doi: 10.1038/s41598-023-40196-4 (PMC10432412; doi:10.1038/s41598-023-40196-4)
Supplement: Supplementary file 1 — Supplementary Tables. [file 41598_2023_40196_MOESM1_ESM.docx]

**Supplementary Table S.1** Correlation matrix of all health-related characteristics with skewed distribution

|  | *chronic* | *recall* | *mobility* | *depressive* | *fruits* |
| --- | --- | --- | --- | --- | --- |
| *chronic* | 1 | -0,18 | 0,49 | 0,3 | 0,01 |
| *recall* | -0,18 | 1 | -0,24 | -0,17 | -0,06 |
| *mobility* | 0,49 | -0,24 | 1 | 0,41 | 0,05 |
| *depressive* | 0,3 | -0,17 | 0,41 | 1 | 0,04 |
| *fruits* | 0,01 | -0,06 | 0,05 | 0,04 | 1 |

Correlation by Spearman’s method; chronic, Number of chronic diseases; recall, Delayed recall; mobility, Mobility limitations index; depressive, Number of depressive symptoms; fruits, Frequency of eating fruits and vegetables per day

**Supplementary Table S.2** Proportion of participants in educational level 0, stratified by sex, age and region

|  | Educational level 0 | Educational level 1-6 |
| --- | --- | --- |
| Female, n (%) | 1906 (4%) | 41052 (96%) |
| Male, n (%) | 1360 (4%) | 33474 (96%) |
| Age, median (IQR) | 73 (17) | 64 (15) |
| *Region* |  |  |
| Western Europe, n (%) | 893 (3%) | 32346 (97%) |
| Southern Europe, n (%) | 2099 (15%) | 11893 (85%) |
| Central and Eastern Europe, n (%) | 235 (1%) | 21285 (99%) |
| Northern Europe, n (%) | 39 (0,4%) | 9002 (99,6%) |
